# The immunomodulatory mechanisms for acupuncture practice

**DOI:** 10.3389/fimmu.2023.1147718

**Published:** 2023-04-06

**Authors:** Meng Wang, Weili Liu, Jiayi Ge, Shenbin Liu

**Affiliations:** State Key Laboratory of Medical Neurobiology, MOE Frontiers Center for Brain Science, Institutes of Brain Science, Huashan Hospital, Fudan University, Shanghai, China

**Keywords:** acupuncture, immune system, neuro-immune regulation, autonomic nervous system, HPA axis

## Abstract

The system physiology approaches that emerge in western countries in recent years echo the holistic view of ancient Traditional Chinese Medicine (TCM) practices that deal with the root, rather than only the symptoms of diseases. Particularly, TCM practices, including acupuncture, emphasize the mobilization of self-healing mechanisms to bring back body homeostasis. Acupuncture has been practiced for over two thousand years to modulate body physiology *via* stimulation at specific body regions (acupoints). With the development of various research on acupuncture therapy, its regulatory effect on the immune system has been gradually recognized, especially on immunological diseases, including infectious and allergic diseases. In this study, we reviewed the immunomodulatory mechanism of acupuncture and systematically integrates existing research to respectively elucidate the modulatory mechanisms of acupuncture on the innate immune system, adaptive immune system, and well-known neuroanatomical mechanisms, including intact somatosensory-autonomic reflex pathway. With the advances made in recent systems physiology studies, we now have a great opportunity to gain insight into how acupuncture modulates immunity, and subsequently improves its efficacy.

## 1 Introduction

Acupuncture practice is one of the most popular forms of alternative and complementary medicine in the world. As a medical practice with roots in China, it has been widely applied for more than three thousand years and has amassed a wealth of clinical and theoretical evidence. Today, acupuncture practice has already received considerable attention. Acupuncture exerts the therapeutic effect *via* stimulating specific sites on the body surface (which we called acupoints). The main methods of stimulation include manual acupuncture, electroacupuncture, and transcutaneous acupoint electrical stimulation. Acupuncture practice is widely used to alleviate or cure various diseases, including endocrine and metabolic diseases, mental and behavioral disorders, neurological disorders, diseases of the circulatory system, cutaneous disorders, diseases of musculoskeletal and connective tissue and so on (1). The whole process is non-invasive and safe. According to recent systematic reviews, acupuncture is a safe and low-cost treatment with minimal side effects. Till now, none of the randomized controlled trials included in these systematic reviews reported life-threatening adverse events (2, 3). With the stable, repeatable, and quantifiable effect, acupuncture practice has become commonly accepted and studied. As early as 1997, the NIH issued a consensus statement on the effectiveness and scientific nature of acupuncture therapy. Until now, more than 3 million American adults have used acupuncture therapy to control chronic pain, which is the most common reason for seeking acupuncture treatment, and the analgesic and anesthetic effects of acupuncture are no longer in doubt (4–6).

Accumulated evidence showed the potential of acupuncture in the treatment of immune system diseases caused by immune system defects. Acupuncture has been demonstrated to regulate the immune system of the body and correct pathological conditions, thereby providing effective relief or treatment of associated diseases. For instance, acupuncture has been shown to modulate systemic inflammatory responses, resulting in a significant improvement in survival rates among mice with fatal sepsis (7). In addition, acupuncture has also been shown to be effective in the treatment of inflammatory bowel syndrome (IBS), mainly by modulating inflammation-related cytokines and immune cell differentiation *in vivo*, reducing inflammatory responses and clinical symptoms (8).

Acupuncture has a promising future as a kind of safe and efficient medical practice to modulate the body’s immune system. Since acupuncture is based on a system that is distinct from modern Western medicine, current research is focusing on the role of acupuncture and the associated neurobiological mechanisms. In recent decades, a series of breakthroughs in the understanding of the modulatory mechanism of acupuncture has been made due to the rapid advancement of biological techniques. The specific neuroregulatory mechanisms of acupuncture are no longer a black box. Herein, we reviewed the immunomodulatory mechanism of acupuncture, and systematically integrated the existing research results to elucidate the regulation of acupuncture on the innate and adaptive immune response, and the well-known neuroanatomy mechanisms of acupuncture in immunomodulation, including intact autonomic reflex pathways. This is of great significance to the popularization of acupuncture as a medical practice.

## 2 Acupuncture modulates innate immune cells’ activity

### 2.1 Mast cells

Mast cells (MCs), are the tissue-resident immune effector cells and the innate immune system’s first line of defense. They react to a range of environmental physical stimuli, such as temperature, pressure, pathogens, trauma, and acupuncture. When exposed to various stimuli, they release strong biological mediators into the tissues around them, triggering an inflammatory reaction that aids in the healing of wounds and the removal of infections (9). MCs are the primary initiators and regulators of inflammatory and other immune responses that are widely distributed in connective tissue, and they largely interact with the external environment close to blood vessels and sensory nerves (10). According to pertinent research, MCs are dispersed along the direction of blood arteries and nerves and enriched close to acupoints in model organisms like rats and humans. To provide analgesic and anti-inflammatory effects, MCs also take part in the nerve transduction process in response to sensory signals from the surface of the body.

The link between the distribution of MCs on the body’s surface and the TCM concept of acupoints can be seen obviously. The acupoints are considered to be dynamic composite structures composed of MCs, nerve fibers, and vascular structures. MCs were found to be densely distributed around the rat acupoints and blood vessels and were visible *via* contemporary methods like histological methods. Electroacupuncture (EA) stimulation encourages the recruitment and migration of MCs along blood vessels and nerves (11, 12). The distribution pattern of MCs was highly correlated with the distribution of 14 classic acupoints in the meridians, suggesting that MCs may be a tissue target of acupuncture and moxibustion stimulation. Li et al. quantified the number and density of skin in different parts of the body using skin biopsies, dermal cell counts, *etc (* 13). Preliminary histological evidence of acupuncture neuro-immunomodulation can be obtained by combining these pertinent investigations.

It is commonly accepted that acupuncture may stimulate afferent neurons to conduct upward and trigger receptors on the body’s surface. Acupuncture can activate MCs, which are a key component of acupoints, through Transient receptor potential vanilloid 2 (TRPV2) channels, which subsequently act on histamine H1 and adenosine A1 receptors to transform mechanical inputs into neurological impulses that go upward (14). The conclusion that MC stabilizers can suppress this neurological communication, provides that at least some of acupuncture’s effects are influenced by MCs activity (11). Following acupuncture, MCs moved to the local acupoints through the blood arteries of the subdermal and subcutaneous tissues, and the aggregated MCs produced more tryptase through degranulation, histamine, and 5-hydroxytryptamine (5-HT) (15–17). The nerve fibers also released more substance P and calcitonin gene-related peptide P (CGRP). Additionally, it has been found that MC activation can result in the production of adenosine triphosphate (ATP), which functions as the primary mediator of acupuncture-induced analgesia through binding to purinoreceptor receptors (18).

It may be presumed that there are connections or information transfers between MCs and nerve terminals that are given the role that MCs play in nerve conduction. The presence of “Synaptic” connections between MCs and nerve terminals has been investigated preliminarily, with immunohistochemistry providing neuroanatomical proof for the control of the neurological immune system (19, 20). Further research is necessary to gain a better understanding of the mechanism of MCs in neuro-immunomodulation induced by acupuncture.

### 2.2 Macrophages

As an essential element of innate immunity, macrophages provide the first line of defense against pathogens (21). Macrophages fulfill their roles by modulating cell division and tissue repair, typically polarizing different phenotypes to obtain distinct functional phenotypes. Traditional macrophage M1 activation and alternative M2 activation are the two extremes of their phenotypic alterations. M1 macrophages release cytokines that prevent neighboring cells from proliferating and breaking down nearby tissues, while M2 macrophages release cytokines that encourage neighboring cells to proliferate and repair nearby tissues. The heterogeneity of the macrophage phenotype is controlled by many physiological and pathological states, as well as environmental influences (22). Studies have revealed that acupuncture primarily controls the polarization of M1/M2 by modifying the production of cytokines related to inflammation and its re-recruitment at the site of inflammatory injury, which improves the anti-inflammatory effect, to reduce inflammatory pain and tissue repair.

Zhang et al. treated the complete Freund’s Adjuvant (CFA)-induced inflammatory pain rats with manual acupuncture (MA), and they found that the inflammatory pain was significantly relieved, and the amounts of macrophages at acupoints were also raised in the experimental group (23). Interleukin (IL)-6, monocyte chemoattractant protein (MCP)-1, and IL-1 are important cytokines that have been identified by statistical and cytokine association networks. These cytokines are also primarily secreted by macrophages (24, 25). This shows that the onset of the analgesic action of MA on inflammatory pain is mediated by macrophages at acupoints. Yang et al. found that Adjuvant-induced arthritics (AIA) had considerable analgesic and anti-inflammatory effects after MA therapy (26). It is believed that the anti-inflammatory effect of MA may be realized by preventing the polarization of the M1 phenotype because M1 macrophages were suppressed, IL-1 levels were decreased, and the immune cell communication network was inhibited at the ST36 (Zusanli, Stomach 36) location.

In addition to anti-inflammatory and analgesic effects, acupuncture has been demonstrated to play a role in tissue repair by regulating macrophages. The recovery phase of muscle injury includes several stages of degeneration, inflammation, regeneration, and fibrosis, with macrophages acting as the primary orchestrator throughout the process (27). Large numbers of macrophages infiltrate the injury site during the post-injury inflammatory phase, and during the post-inflammatory tissue regeneration phase, macrophages control tissue repair by flipping to distinct phenotypes (28). Yan et al. in establishing a rat model of skeletal muscle injury, EA was applied to ST36 (29). The findings revealed that the EA group’s secretion of IL-4, IL-13, and interferon (IFN)-γ was greatly enhanced. IFN-γ was secreted less often, and there were more M2-type macrophages present, which expedites the repair of skeletal muscle injury.

As previously mentioned, macrophages control the inflammatory-anti-inflammatory balance *in vivo* through phenotypic flipping, as was previously mentioned. In a study by Hu et al. (30), Low-frequency electrical stimulation (LFES) of GB34 (Yanglingquan, Gallbladder 34) and ST36 acupoints helped prevent muscle atrophy by generating a brief inflammatory response in a rat model of CKD-induced skeletal muscle atrophy. After day 3, pro-inflammatory M1 macrophage levels in muscle tissue started to decline during the early phase of stimulation, whereas anti-inflammatory M2 macrophage levels started to rise. Acupuncture may play a role in preserving homeostasis by controlling macrophages. Since insulin-like growth factor (IGF)-1, which is mostly produced by macrophages, is up-regulated to enhance protein metabolism and encourage the synthesis of muscle.

### 2.3 Neutrophils

The neutrophil is myeloid leukocytes and is one of the main effector cells of the innate immune response. It can regulate the body’s immune response, such as acute injury and repair process, chronic inflammation process, and so on, mainly through the recruitment and infiltration of infected and damaged tissues, recognition and phagocytosis of microorganisms, killing pathogens, and making a difference (31). Therefore, the enrichment of neutrophils is often regarded as a marker and biological indicator of acute infection injury. Today, in addition to its role in acute infection, the role of neutrophils in the maintenance of immune homeostasis has also been much studied (32).

Neutrophils are produced daily in the bone marrow and makeup 50-70% of all circulating white blood cells in humans. They are synthesized and converted rapidly, especially during periods of inflammation and immune imbalance in the body (33). So, when acupuncture regulates the immune function of the body, it is often accompanied by the regulation of the quantity and activity of neutrophils. A small clinical study showed that acupuncture can improve the immune protection of ovarian cancer patients due to chemotherapy-induced immune suppression caused by the reduction of white blood cell count (34). Another animal study found that acupuncture significantly improved survival in septic rats, by reversing the migration of neutrophil injury to the peritoneal cavity (35). Zhang et al. demonstrated that, in a mouse model of left anterior descending (LAD) coronary artery ligation to mimic myocardial injury, acupuncture inhibited the activation of the NLRP3 inflammasome, promoting macrophage M2 polarization, and reducing the recruitment of neutrophils in the damaged myocardium, thereby reducing infarct size and improving cardiac function (36).

### 2.4 Natural killer (NK) cells

NK cells, the body’s third lymphocyte after T and B cells, are the primary effector cells in the innate immune system. They can immediately release a variety of cytokines and chemokines, directly and non-specifically killing tumors and other aberrant cells as well as aging cells. NK cell-mediated immunotherapy has become a focus of research for the treatment of cancer and other immunosuppressive diseases due to its potent cytotoxic effects on aberrant cells in the body (37, 38). NK cells are generally quiescent under normal physiological settings, but when activated, they can infiltrate tissues and release cytotoxic granules containing perforin and granzyme, which directly lyse aberrant cells and have a killing effect (39). IFN, tumor necrosis factor (TNF), and other chemokines and cytokines that control the adaptive immune response can be secreted by NK cells at the same time.

Prior studies have shown that acupuncture therapy can significantly boost NK cell activity, regulate the proportion of NK cell subsets, and trigger the release of related cytokines, resulting in analgesia, regulation of the stress response, relief from symptoms of morphine withdrawal and immunosuppressive effects brought on by cyclophosphamide, *etc (* 40–43). According to Yu et al., EA stimulation of the bilateral ST36 acupoints dramatically elevated the toxicity of NK cells in the rat spleen (44). Other research revealed that EA stimulation of the ST36 acupoint significantly increased the levels of IL-2 and IFN-γ in the spleen. Cytokine levels were also significantly positively correlated with the toxicity of splenic NK cells, with endogenous IFN-γ playing a significant role in the EA’s intervention in the activation of NK Cells (43).

So, how does acupuncture affect the quantity and activity of NK cells in the spleen? According to Ham et al., destroying the lateral hypothalamus can decrease the activity of NK cells, and electroacupuncture ST36 can reverse this effect, revealing that the lateral hypothalamic area (LHA) plays an important role in the neuroimmunomodulation of NK cells by acupuncture. The use of a lateral hypothalamic injury rat model by Choi et al. also provides evidence to support these finding (45, 46). When β-endorphin travels *via* the circulation to lymphoid tissues like the spleen, it binds to opioid receptors found on the surface of NK cells and facilitates the activation of NK cells by controlling the production and distribution of IFN-γ (44, 47). However, it is significant to note that LHA damage does not entirely reverse the effects of EA, indicating that the regulation mechanism of EA stimulation on NK cells may be more intricate than previously assumed and additional research is required (46).

### 2.5 Astrocytes and microglia

Astrocytes are neuroglia derived from neuroectoderm and neural progenitors, which are abundant in the central nervous system (CNS) and play essential functions in the CNS of healthy and diseased organisms. During CNS injury or infection, many changes in astrocytes’ morphology, molecular expression, and function affect the progress of various CNS diseases (48). The microglia are a type of important immune cell in the CNS. It is similar to tissue-resident macrophages in molecular morphology and biology. It can be divided into pro-inflammatory type M1 and anti-inflammatory type M2. The dual immunomodulatory effect of which is mainly realized through the transformation of its phenotype (49). In the early stages of injury, M1 is polarized, secreting high levels of pro-inflammatory factors such as IL-1β, IL-6, TNF-α, *etc.* to initiate immune defense. Therefore, astrocytes and microglia are important players in many neurological disorders, regulating neuroinflammatory responses (48–50).

Previous studies have shown that acupuncture can achieve anti-inflammatory and neuroprotective effects by modulating the inflammation of the nervous system caused by neurological disorders such as Alzheimer’s disease (AD), Parkinson’s disease (PD), traumatic brain injury (TBI), spinal cord injury (SCI), *etc.*, alleviating the pathological reaction of the disease. In cerebral ischemia-reperfusion disease, inflammatory responses are activated, microglia are activated, and large numbers of circulating inflammatory cells infiltrate the damaged areas, leading to the secretion of inflammatory-associated factors and a cascade of responses that ultimately lead to secondary brain damage (51). EA at LI11 (Quchi, Large intestine 11) and ST36 acupoints of MCAO rats for 3 consecutive days effectively reduced the infarct volume and neurological deficit and improved the velocity, balance, and coordination of motor function, and the number of BrdU/NEUN double-positive cells and striatum double-positive cells in the brain of EA rats was significantly increased, suggesting that EA may achieve the neuroprotective effect by inducing cells to proliferate and differentiate into astrocyte and mature neurons (52). Another study found that EA of PC6 (Neiguan, Pericardium 6) and LI11 ameliorated nerve injury and reduce inflammation, which may be related to the inhibition of the activity of the toll-like receptor (TLR)4-nuclear factor kappa-B (NF-κB) signaling pathway in microglia (53). According to Liu et al., EA ameliorated the degeneration and necrosis of the ischemic penumbra cortical microglia, inhibited the transformation of the microglia to the M1 proinflammatory phenotype, and downregulated the expression of Iba-1 and CD11b. In addition, it also inhibited the expression of NF-κB, IL-1β, and TNF-α (54).

For neurodegeneration conditions such as PD or AD, the activation of microglia and astrocytes is an important pathological feature (55). In addition, the inflammatory environment can enhance the aggregation of pathogenic α-synuclein, further activate the aggregation and proliferation of microglia, induce pathological cascade reaction, and aggravate the disease progression. Therefore, the regulation of microglia and astrocytes has important clinical significance in disease control (56).

Acupuncture can enhance the structure and performance of synapses, activate astroglia and microglia, foster Neuroplasticity, and ameliorate the symptoms of neurological deficits (57). In a mouse model of PD, acupuncture treatment reduced the activation of microglia and astrocyte, and inhibited the neuroinflammatory response associated with the PD phenotype (58). The activation of glial cells in hippocampal CA1 and DG regions and the polarization regulation of M2 microglia were observed in AD rats treated with EA, and increased levels of anti-inflammatory cytokines (IL-4 and IL-10) and decreased levels of Proinflammatory cytokine (TNF-α, IL-1β, and IL-6) may be associated with NF-κB and STAT6 pathways (59).

According to extant studies, the mechanism of EA inhibition of glial cell activation is associated with sensory neurotransmission in the dorsal root ganglia (DRG). EA has been demonstrated to reduce neuropathic pain by inhibiting Toll-like receptor 4 (TLR4) signaling in DRG neurons, while concurrently upregulating transient receptor potential vanilloid 1 (TRPV1) to attenuate spinal glial activation (60).

## 3 Acupuncture-induced modulation of the adaptive immune response

### 3.1 Th1/Thbalance and Th17/Treg balance

As one of the most significant subsets of peripheral lymphocytes, CD4^+^ T helper (Th) cells affect both innate and adaptive immune responses by producing cytokines and interacting with other cells (61). Subsets of CD4^+^ T cells include Th1 cells, Th2 cells, Th9 cells, T follicular helper cells (Tfh cells), Th17 cells, Treg cells, and so on. Th1 cells mediate cellular immunity or delayed hypersensitivity (DTH) and promote the inflammatory reaction. They mainly secrete IL-2, IL-6, IL-12, IFN-γ and TNF-α. Th2 cells mediate humoral immunity, inhibit the inflammatory reaction, and mainly secrete IL-4, IL-5, and IL-10 (62). In a normal physiological state, Th1/Th2 maintain a certain balance and jointly maintain the balance of immune function. The imbalance of Th1/Th2 cells may be the main cause or pathological outcome of some infectious or allergic diseases (63).

In addition to Th1 and Th2, Th17 cells are a third crucial subset of CD4^+^ T helper cells. By producing IL-17 and other Proinflammatory cytokines like TNF-α, IL-6, IL-22, and IL-21, then induced neutrophils migrate to the site of infection in the body, causing an inflammatory response that is crucial for the emergence of autoimmunity (64). Regulatory T (Treg) cells are FOXP3-expressing immune cells that are naturally present in the immune system. It suppresses the immune response of effector cells by secreting TGF-β1 and/or IL-10, thereby inhibiting the proliferation of T helper cells and the production of inflammatory factors, regulating the homeostasis of the body and preventing autoimmune diseases (65). There is a crucial plasticity between Th17/Treg cells, which maintains the immune balance of the body. In inflammatory and autoimmune diseases, the Th17/Treg balance is broken, which promotes the initiation and maintenance of inflammation (66).

Asthma is a severe chronic respiratory disease characterized by airway hyper-responsiveness, airway inflammation, and airway remodeling. The immune response of CD4^+^ T cells and the regulation of cytokines secreted by CD4^+^ T cells are the key detrimental changes within the pathological progression (67). Th2 and Th17 cells and their cytokines promote the exacerbation of asthma symptoms, while the regulation of Th1 and Treg cells can suppress the pathological symptoms of asthma. Therefore, the regulation of Th1/Th2 and Th17/Treg balance can alleviate the symptoms of asthma and provide a therapeutic effect. To date, there exist several animal experiments and clinical studies to verify the effect of acupuncture treatment on asthma, mainly by regulating the number and activity of CD4^+^ T cells to play an anti-inflammatory role (68). Zhao et al. found that after using ovalbumin to induce mouse asthma models and acupuncture at GV14 (Dazhui, Governing vessel 14), BL13 (Feishu, Urinary bladder 13), and ST36 acupoints, the levels of IFN-γ in blood and bronchoalveolar lavage fluid(BALF) were increased and the levels of IL-4, IL-17, and TGF-β were decreased in the treatment group as compared with the control group, on CD4^+^ T cells, acupuncture corrected the imbalance of Th1/Th2 and Treg/Th17 cells (69). Consistent with this conclusion, Dong et al. observed that after acupuncture treatment of ovalbumin-induced asthma in mice, the activities of Th1 and Treg cells were enhanced (70).

In addition to asthma, acupuncture can be effective in treating IBS and other diseases by regulating the differentiation of CD4^+^ T cells. In a collagen-induced rat model of arthritis, EA can reestablish the balance of Th17/Treg cells and relieve inflammation of arthritis (71). In the ulcerative colitis (UC) mice model group, the expression of TLR2 and TLR4 in the splenic lymphocytes was up-regulated, and the symptoms of UC were significantly alleviated after EA stimulation of ST36 (72). The immunosuppression after surgical trauma can also be effectively relieved by EA. The balance between Th1 and Th2 cytokines in the spleen was detected after acupuncture, suggesting the pathway of EA relieving immunosuppression (73).

### 3.2 CD8^+^ T cells differentiation

CD8^+^ T cells are a subset of cytotoxic T cells that are essential for both tumor and viral defense (74). It can differentiate into effector and memory T cells that function to help mediate pathogen clearance and provide long-term protective immunity (75). In antiviral infection, which is mainly mediated by CD8^+^ T cells, other immune cells and cytokines have contributed to it. In the presence of persistent antigens created by ongoing infection, CD4^+^ T cells can prevent CD8^+^ T cells from developing tolerance, and they can also encourage the recruitment, proliferation, survival, and exerting actions of CD8^+^ T cells in a pathogenic environment (72).

Acupuncture maintains the homeostasis of the body by regulating the differentiation of T cells and the ratio of different lymphocyte subsets. According to Yamaguchi et al., the number of CD2, CD4, CD8, CD11b, CD16, and CD56 cells and the levels of IL-4, IL-1β, and IFN-γin human peripheral serum cells increased significantly after acupuncture (42). Another clinical study showed that after acupuncture, the serum total IgE level in patients with allergic asthma decreased significantly, showing a good therapeutic effect. At the same time, CD3^+^, CD4^+^, and CD8^+^ T lymphocytes in peripheral blood were dramatically increased, indicating that acupuncture may be mediated by CD8^+^ T cells (68). In a mouse model of collagen-induced arthritis (CIA), however, the CD4^+^/CD8^+^ ratio was maintained in a near-normal range in acupuncture compared with the control group, with effective suppression of inflammatory responses (54). For sepsis mice, EA could increase the number of CD3^+^ T cells, maintain the ratio of CD4^+^/CD8^+^ T cells and protect the intestinal mucosal immune barrier (76).

## 4 Somatosensory-autonomic reflex to modulate the immune response

### 4.1 Neuroanatomical basis of somatosensory mechanisms for acupuncture-induced immunomodulation

Many of the acupoints used in acupuncture have neuroanatomy implications consistent with Western medical concepts, and acupuncture works on the body through several mechanisms, even though its therapy is based on a completely different set of systems from Western medicine. With the development of new technologies such as molecular and cell biology, our understanding of this practice is deepening. Today, researchers can use various research methods to develop and enhance this medical practice, thanks to some ground-breaking studies in recent years that have established a research paradigm to explain its neuroanatomy mechanism. Acupuncture acts as a non-nociceptive mechanical stimulus, activating a variety of mechanosensitive sensory neurons on the skin’s surface. The molecular signals of the mechanical stimulus are then transduced through primary sensory afferent nerve fibers, projecting to the interneurons of the spinal cord, brainstem, and hypothalamus. These signals are then sent to the corresponding neural pathways *via* efferent nerves, regulating the body’s feeling, movement, and more (77, 78).

There are several types of afferent fiber, thick myelinated Aα and Aβ (group I and II), thin myelinated Aδ (group III), and thinner unmyelinated C (group IV) fibers (79). These fibers, which are mostly located in the trigeminal ganglion (TG) and dorsal root ganglia (DRG), contain peripheral axon branching at the terminals of nociceptive receptors that gather and convey impulses to the surface of the body. The physical basis for the specificity, intensity and frequency dependence of acupuncture points may be the many types of activated peripheral afferent nerve fibers (77). The frequency of EA stimulation, the position, and the strength of the acupuncture will all have a varied impact (80–82). Xin et al. found that the segmental analgesia of EA at ST36 with lower intensity is partially mediated by ASIC3 receptor on Aβ-fiber, whereas systemic analgesia induced by EA with higher intensity is more likely induced by TRPV1 receptor on Aδ- and C-fibers (83). This is of great significance to the clinical application of acupuncture treatment.

The autonomous nervous system (ANS) is essential in maintaining the stability of the organism. It is composed mainly of the sympathetic and parasympathetic nerves and plays a significant role in acupuncture’s therapeutic effects on gastrointestinal motility, inflammation, analgesic anesthesia,and more (84). However, recent studies have demonstrated that acupuncture’s autonomic regulation is not as straightforward as that of the sympathetic or parasympathetic pathways. The functions of the sympathetic nervous system and parasympathetic nervous system are not mutually exclusive, and in many cases, they collaborate to combat disease (85). Here we provide a brief review of several acupuncture-regulated neuro-reflex pathways that have been discovered in recent years, such as the vagal-adrenal pathway, the cholinergic anti-inflammatory pathway (CAIP), the spinal sympathetic pathway, and the involvement of the brain-gut axis (BGA).

### 4.2 The vagal-adrenal anti-inflammatory pathway

The recently discovered vagal-adrenal pathway is a signal pathway through which EA stimulation of ST36 acupoint can exert an anti-inflammatory effect. Torres-Rosas et al. found that EA stimulation of ST36 could effectively inhibit systemic inflammation and improve survival in LPS- and cecal ligation and puncture (CLP)-induced septic mice. Vagotomy and adrenalectomy could block the anti-inflammatory effect of EA, they, therefore, speculate that EA may act through the vagal-adrenal pathway. After EA, the levels of catecholamines, especially dopamine, mainly released from the adrenal medulla were dramatically increased. But only after dopamine was blocked by an inhibitor, the anti-inflammatory effect of EA was blocked. And the use of dopaminergic agonists can mimic the effects of EA and control inflammation in mice with adrenal insufficiency sepsis (86). This demonstrates the existence and stabilization of the vagal-adrenal signaling pathway.

Further, Liu et al. used genetic manipulation strategy demonstrating that NPY^DBH+^ chromaffin cells are involved in this anti-inflammatory axis (7). It was then shown that low-intensity EA stimulation (0.5 mA) with PROKR2-Cre^+^ sensory neurons dependent can evoke this vagal–adrenal axis from the hindlimb ST36 acupoint, but not from the abdominal ST25 acupoint. Even high-intensity EA stimulation (3 mA) of ST25 failed to activate vagal parasympathetic efferent neurons located in DMV, demonstrating the acupoint selectivity in driving vagal reflexes. Activation of this vagal–adrenal axis sufficiently attenuates LPS- and CLP-induced systemic inflammation and protects against septic death in mice, and notably, this reflex operates in a disease state-independent manner, producing anti-inflammatory effects irrespective of EA stimulation conducted before or after cytokine storm has reached peak levels. Thus, EA stimulation can drive this pathway and modulate systemic inflammation in a manner dependent on acupoint selection, and stimulation intensity.

### 4.3 The cholinergic anti-inflammatory pathway

As a neuro-immunomodulatory mechanism regulated by the vagus nerve, CAIP is responsible for controlling the body’s inflammatory response and has a pronounced inhibiting effect on both local and systemic inflammation (87–89). Acetylcholine (Ach) is the primary neurotransmitter of the parasympathetic nervous system, and it can be received by muscarinic and nicotine receptors expressed by immune cells (90). In CAIP, the nicotinic acetylcholine receptor type 7 subunit (α7-nAChR) binds the majority of the Ach released from the vagus nerve, which prevents NF-κB nuclear translocating and prevents monocyte and macrophage cytokine releasing. Regulation of immune responses (91, 92) may be useful in treating or reducing immune disequilibrium disorders like inflammatory bowel disease and osteoarthritis (93, 94).

Vagus nerve stimulation has emerged as a promising therapeutic approach in recent years, activating anti-inflammatory pathways through cervical and trans-auricular electrical stimulations (95–97). However, this treatment requires invasive surgery, with some associated risk (98). Acupuncture at specific acupoints also can effectively activate the vagus nerve and achieve anti-inflammatory effects through CAIP (99, 100). By contrast, its non-invasive nature and low risk make it an ideal alternative to inflammatory therapy.

Song et al. found that CAIP activation played an important role in the process of EA stimulation of ST36 to inhibit the inflammatory response in septic rats. Suppressed inflammatory responses are exacerbated after the bilateral vagal blockade, and similarly, ST36 EA no longer has an anti-inflammatory effect after the use of the α7-nAchR antagonist α-BGT (101). Consistent with the results of this study, EA stimulation of Li4 (Hegu) can also significantly inhibit the inflammatory reaction in endotoxemia rats and improve the survival rate. The spleen is an important organ in this anti-inflammatory pathway. The vagus nerve terminates in a synaptic-like structure around the main cells of the celiac-superior mesenteric plexus, where the catecholaminergic splenic fibers originate. EA at Li4 can activate the vagus nerve and increase the noradrenaline released by the spleen. The adrenergic receptor, which is expressed in B and T cells of the spleen, produces acetylcholine, and anti-inflammatory effects are achieved by activating the nicotinic acetylcholine receptor expressed on macrophages and inhibiting the release of related pro-inflammatory factors (100).

Besides systemic inflammation, acupuncture at specific acupoints can also exert its effect on local inflammation through CAIP. In the chronic obstructive pulmonary disease (COPD) rat model, acupuncture at ST36 and BL13 can down-regulate the levels of inflammatory cytokines, reduce the inflammatory reaction of lung tissue and improve lung function. Furthermore, the enhancement of the cervical vagus nerve discharge signal was observed in the experiment, and the effect of EA was blocked after antagonizing the action of α7-nAChR with α-BGT (102). EA stimulation of ST36 could inhibit the inflammatory reaction and then reduce the infiltration of white blood cells in the mice model of acute pancreatitis. This regulatory effect can be blocked by vagotomy and α-BGT, suggesting an important role of CAIP in this process (103).

### 4.4 The spinal-sympathetic pathway

As described above, high-intensity EA stimulation of ST25 does not activate the vagal-adrenal axis but rather drives the spinal cord sympathetic pathway. Meanwhile, Liu et al. and other studies have shown that EA at ST25 can also activate the spinal-sympathetic pathway and have an anti-inflammatory effect (104). Axons from sympathetic ganglion neurons of the spinal cord enter the adrenal gland and terminate in the cells of the adrenal medulla globular zone, releasing catecholamine and neuropeptide Y (NPY), suggesting a direct sympathetic nervous system of the adrenal medulla. After stimulation of the spinal sympathetic pathway, sympathetic nervous system control promotes the synthesis and secretion of noradrenaline in the adrenal medulla, which further activates the β2-adrenergic receptor of splenic cells and regulates the activation of inflammatory cells, the secretion of pro-inflammatory and anti-inflammatory factors, produces anti-inflammatory effects.

The spinal sympathetic pathway can be activated by acupuncture at multiple acupoints. In LPS-induced septic mice, EA at ST25 of 3 mA significantly decreased proinflammatory cytokine IL-6 and IL-1β, and up-regulated the anti-inflammatory cytokine IL-10, increasing the survival rate of septic mice. Another study used 1 Hz low-frequency EA to stimulate the ST36 acupoint and found that sympathetic ganglion neurons were activated and acted on the β-adrenergic receptor of immune cells to suppress the yeast polysaccharide-induced peripheral inflammatory response (105). EA with Li4 can significantly inhibit systemic inflammation and improve the survival rate in LPS-induced lethal sepsis rats. This specific anti-inflammatory effect requires activation of the sympathetic nervous system (100). After sympathectomy, the anti-inflammatory effect was significantly suppressed when 6-hydroxy dopamine (6-OHDA) was used, indicating that the sympathetic nerve is involved in mediating the anti-inflammatory effects.

### 4.5 The brain-gut pathway

The bidirectional communication and coordination between the brain and the intestine are mainly mediated by the brain-gut axis (BGA) (106). The role of the BGA in the maintenance of homeostasis has been extensively studied over the past decade. It is crucial for preserving homeostasis in the organism, particularly that of the immune system (107). The brain-gut signaling pathway mainly involved the central nervous system (CNS), the enteric nervous system (ENS), the autonomic nervous system (ANS), the hypothalamus-pituitary-adrenal (HPA) axis, and others. These pathways regulate the gut microbiota, brain-gut peptides, local immune systems in the gut, and so on. Through the BGA, signals from the brain affect the body’s sensory, motor, and gut microbiota. In contrast, gut microbiota and peptides can further influence brain function (108). This pathway plays an important role in the pathology of neurodevelopmental disorders, neurodegeneration, gastrointestinal disorders, and others, which is widely considered a novel therapeutic target for disease (108–111).

IBS is a widely recognized functional gastrointestinal disorder, which is often seen as a pathological consequence of BGA disease (111). In addition to typical symptoms such as abdominal pain, abdominal distention, intestinal dysfunction, low-grade inflammation, and local immune activation, something else has been observed in the intestinal wall of patients, which may lead to neurological dysfunction and impaired immune system within the intestinal mucosa (112). Acupuncture has been used to treat gastrointestinal disorders for millennia, and its regulatory role in IBS and other gastrointestinal disorders has been confirmed by several clinical and basic studies (113, 114). Sun et al. found that acupuncture at acupoints ST36 and ST25 in IBS mice alleviated IBS-like symptoms and significantly reduced the content of 5-hydroxytryptamine (5-HT) and CGRP in BGA, increasing the content of NPY (115). Another study showed that EA could decrease the expression of CRF and CRF-R1 in the hypothalamus of IBS rats, relieve anxiety and depression, decrease the expression of CRF-R1 in the gastrointestinal mucosa, and increase the expression of ZO-1. Regulation of the tight junction, which repairs the intestinal mucosal barrier, suggested a potential dual therapeutic role for EA in the regulation of disturbed gut-brain interactions in IBS rats (116).

In another study conducted by song et al. in the post-inflammatory irritable bowel syndrome (PI-IBS) model using trinitrobenzene sulfonic acid (TNBS) induction, electroacupuncture stimulation of ST25 and ST36 acupoints could have a positive effect on alleviating visceral allergy symptoms and protecting the intestinal mucosa by changing the number and type of microflora in the intestine (117).

In this process, acupuncture, a non-invasive nerve stimulus, may control intestinal inflammation and the secretion of related neurotransmitters through a somatosensory-autonomic reflex pathway, thereby restoring the balance of BGA (118). Alterations in the gut microbiota and signals of immune activation can be transmitted to the brain *via* the vagus or sympathetic nerves, thus establishing a bidirectional brain-gut regulation (119). However, further exploration of the specific regulatory pathway is needed.

## 5 HPA axis involved with immunoregulation by acupuncture

The hypothalamus-pituitary-adrenal axis (HPA axis) appears to be one of the most significant stress reactions and is crucial for energizing and re-establishing the body’s equilibrium. The HPA axis is triggered by multiple stimuli, including physiological, psychological, and immunological, which promote the synthesis and release of glucocorticoid and other hormones throughout the body (120). According to relevant studies, the autonomic nervous system plays a role in the regulation of the HPA axis (121). The involvement of the HPA axis in the regulation of immune diseases by acupuncture indicates the fine regulation of the nerve-endocrine-immune network by acupuncture.

Zhang et al. found that in a mouse model of CFA-induced inflammation, EA at 10 Hz suppressed peripheral inflammation by activating the HPA axis and the nervous system, whereas adrenalectomy was performed to block the HPA axis (10), there was no significant decrease in inflammation levels relative to the control group (122). For an OVA-induced asthma mouse model, acupuncture was demonstrated to effectively inhibited airway hyperresponsiveness, reduce lymphocyte count in BALF, attenuate airway inflammation and secretion of associated inflammatory cytokines, increase levels of corticotropin and cortisol in plasma, suggesting that the activity of the HPA axis may be implicated in the immune regulation of airway inflammation (123). Acupuncture can regulate the homeostasis of the body by regulating the HPA axis, inhibiting and alleviating the inflammatory response that may be induced by stress (124).

## 6 Perspectives and conclusions

Currently, some human experiments suggest that there is no significant difference in therapeutic effect between the acupuncture group and the sham-acupuncture group (which was designed by attaching a blunt to the epidermis instead of inserting the skin), leading to the conclusion that the acupuncture effect is negated. However, it is important to note that the sham needle group did not undergo complete control in the experimental design. There are a large number of c-low threshold mechanical receptors and nonpeptidergic sensory nerve fibers innervate the epidermis, and these receptors somehow be activated by blunt needle and then cause a response. Additionally, there have been numerous animal experimental studies to explore the physiological mechanism of acupuncture, which provides a solid theoretical basis for acupuncture therapy. Therefore, the results of the current human experimental design are not sufficient to conclusively disprove the efficacy of acupuncture treatment.

**Figure 1:**
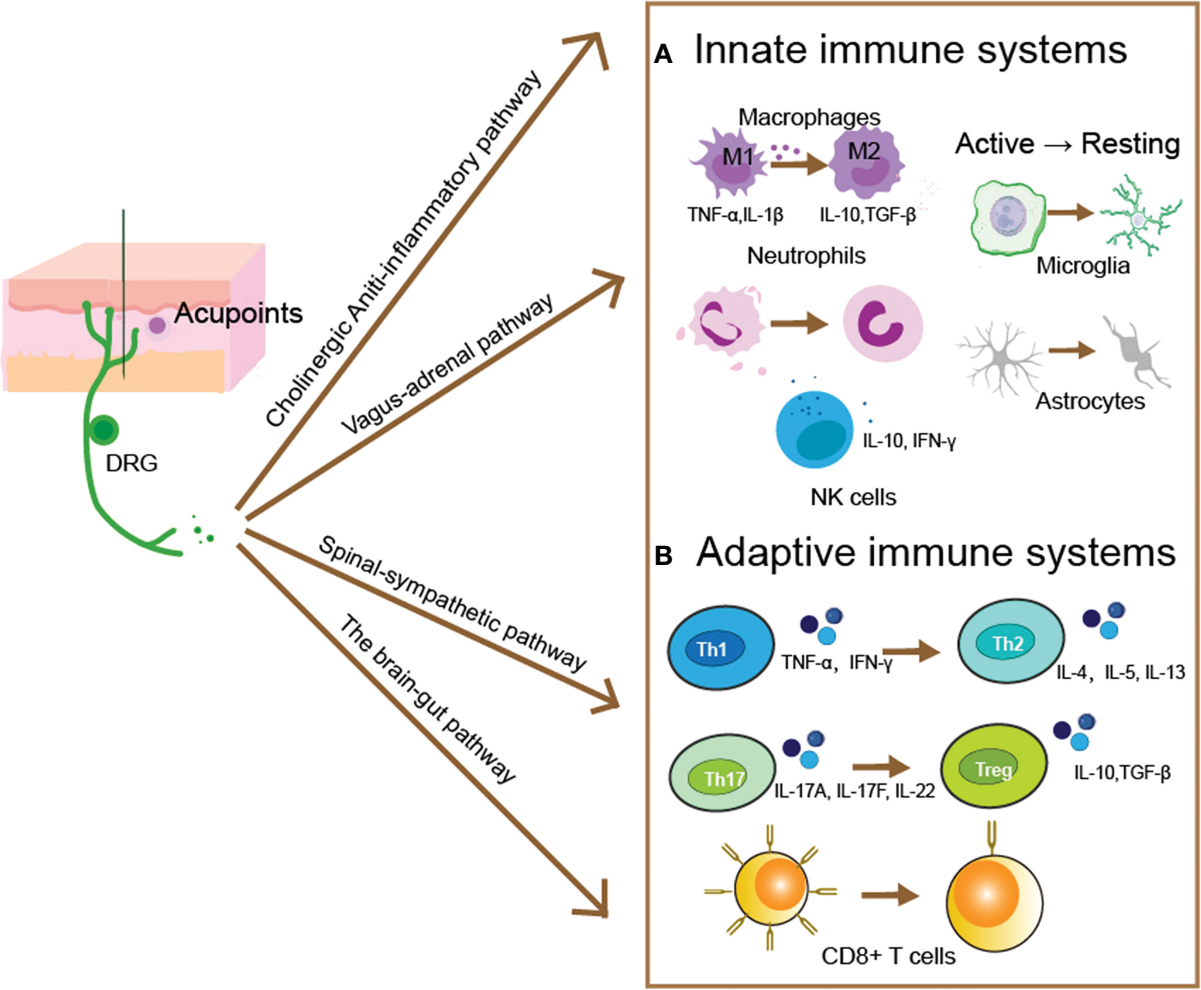
Acupuncture evoked pathways for modulating innate and adaptive immune responses. After acupuncture evoked a mechanical and thermal stimulus within acupoints, the generated electrical signal then transmitted by somatic sensory fibers to the central nervous system, and produced distinct descending regulatory pathways. Such as the cholinergic anti-inflammatory pathway, the vagal-adrenal pathway, the spinal-sympathetic pathway, and the brain-gut pathway. These pathways then to restoring the immune homeostasis by moderating innate **(A)** and adaptive **(B)** immune responses. **(A)** Acupuncture modulates the innate immune response by regulating innate immune cells’ activity and the capacity of release cytokines. The proliferation and differentiation of pro-inflammatory M1 macrophages can be blocked by acupuncture, whereas the number of anti-inflammatory M2 phenotypes is increased, along with the secretion of anti-inflammatory cytokines such as IL-10 and TNF-β. Acupuncture can regulate the quantity and activity of neutrophils and reverse the migration of neutrophils to the site of inflammation. NK cells can release cytotoxic granules containing perforin and granzyme to Lyse and kill abnormal cells, and acupuncture can increase the number and activity of NK cells. In the central nervous system, astrocytes and microglia can be modulated by acupuncture to reverse the activated state, and reduce the inflammatory responses. **(B)** Acupuncture primarily controls the adaptive immune system by controlling the development of T cells, which produces CD4^+^ T cells for humoral immunity and CD8^+^ T cells for antiviral immunity. The balance between pro-inflammatory Tregs and anti-inflammatory Th17, as well as between pro-inflammatory Th1 and anti-inflammatory Th2, is regulated by acupuncture. Maintaining the ratio of CD4^+^/CD8^+^ T cells while controlling the quantity and activity of CD8^+^ T cells will help the body’s immune homeostasis.

In this review, we incorporated and covered the immunomodulatory function and mechanism of acupuncture practice. In addition to dramatically regulating immune cells and molecules, including innate and adaptive immune responses, acupuncture can stimulate and support immunological responses that are anti-inflammatory and anti-infectious. It then discusses how acupuncture controls the immune response. A particular conduction pathway is used by the mechanical stimulation signal of acupuncture to affect immunological organs and cells (See **Figure 1**). It is essential to promote the use of acupuncture as a scientific medical practice, to some extent, led to a breakthrough in our understanding of the effects and characteristics of acupuncture therapy. Despite the numerous advances in the field of acupuncture, there remain many challenges. The regulatory effect of acupuncture on the body often acts on multiple systems simultaneously, such as the regulation of the neural-endocrine-immune network. This is in line with the holistic perspective of TCM, which suggests that the therapeutic effect of acupuncture practice is frequently the result of the integration of a multi-system network. It is now challenging to properly comprehend the regulatory impact of the system on the organism because the majority of related mechanism research is concentrated on a single system and the outcomes are too dispersed. Consequently, further research is needed to ascertain how acupuncture affects the body’s immune system as a whole.

Medical research in western countries has largely relied on reductionist approaches, which seek to understand the individual components of a complex system at the molecular level. While this approach has been successful in managing associated symptoms, it has not been as successful in treating complex diseases, such as sepsis, severe arthritis, gastrointestinal disorders and neurological disorders. Because complex diseases involve many redundant molecular and cellular pathways, making it difficult to target them all. The development of system physiology offers potential solutions to this problem. System physiological research focuses on elucidating the interplay between various systems in the disease process, such as the dynamic interaction between neuro-immune-target tissues. This approach echoes the holistic view of ancient acupuncture practices, which emphasize the mobilization of self-healing mechanisms to restore body homeostasis, rather than just treating the symptoms. With the advances made in recent systems physiology studies, we now have a great opportunity to gain insight into how acupuncture works to modulate immunity, and subsequently improve its efficacy.

## Author contributions

MW wrote the original draft. WL, JG and SL edited the manuscript. All authors contributed to the article and approved the submitted version.
